# Effect of Industrial Byproduct Gypsum on the Mechanical Properties and Stabilization of Hazardous Elements of Cementitious Materials: A Review

**DOI:** 10.3390/ma17174183

**Published:** 2024-08-23

**Authors:** Pengfei Wu, Xinyue Liu, Xiaoming Liu, Zengqi Zhang, Chao Wei

**Affiliations:** 1 State Key Laboratory of Advanced Metallurgy, University of Science and Technology Beijing, Beijing 100083, China; 15232859538@163.com (P.W.); b20200134@xs.ustb.edu.cn (X.L.); weichao0810@126.com (C.W.); 2 School of Metallurgical and Ecological Engineering, University of Science and Technology Beijing, Beijing 100083, China

**Keywords:** industrial solid waste, mechanical properties, stabilization of hazardous elements, low-carbon, sustainable development

## Abstract

**Highlights:**

1. Data analysis revealed that an appropriate amount of BPG can enhance the mechanical properties of cementitious materials, with compressive strength increasing by an average of 7–30%.
2. The mechanical properties and underlying mechanisms of BPG-based cementitious materials were summarized.
3. This study analyzed the mechanisms of the conversion and utilization of elements of impurities in BPG, as well as the solidification mechanisms of hazardous elements.
4. The mechanisms of synergistic utilization between BPG and silica–alumina-based solid waste, as well as alkaline solid waste, are summarized, showcasing innovative waste management strategies.
5. A sustainable solution for promoting the collaborative low-carbon development of various industrial solid wastes using BPG is proposed.

**Abstract:**

Industrial byproduct gypsum (BPG) is a secondary product that is mainly composed of calcium sulfate discharged during industrial production. BPG primarily consists of desulfurized gypsum, phosphogypsum, and titanium gypsum, which account for 88% of the total BPG in China. The large-scale utilization of these three types of solid waste is crucial for the safe disposal of BPG. BPG contains various impurities and harmful elements, limiting its applications. The continuous accumulation of BPG poses a serious threat to the safety of the environment. Based on a literature review (2021–2023), it was found that 52% of BPG is used in the preparation of cementitious materials, and the addition of BPG results in an average improvement of 7–30% in the mechanical properties of cementitious materials. Moreover, BPG has a positive impact on the immobilization of hazardous elements in raw materials. Therefore, the utilization of BPG in cementitious materials is beneficial for its large-scale disposal. This study primarily reviews the effects and mechanisms of BPG on the mechanical properties of cementitious materials and the solidification of hazardous elements. Most importantly, the review reveals that BPG positively influences the hydration activity of silica–alumina-based solid waste (such as steel slag and blast furnace slag) and alkaline solid waste (such as carbide slag and red mud). This improves the proportion of solid waste in cement and reduces production costs and carbon emissions. Finally, this article summarizes and proposes the application of BPG in cementitious materials. The application of BPG + silica–alumina solid waste + alkaline solid-waste-based cementitious materials is expected to realize a new type of green ecological chain for the joint utilization of multiple industrial solid wastes and to promote the low-carbon sustainable development of industrial clusters.

## 1. Introduction

**Figure 1:**
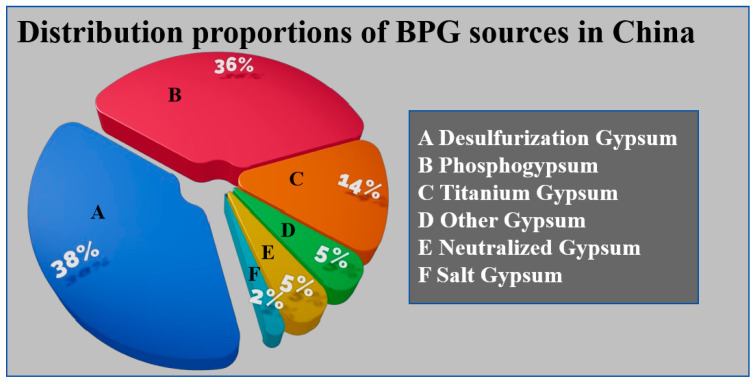
Distribution proportions of BPG sources in China [6]. Note: This figure is a summary diagram and does not involve copyright issues.

**Figure 2:**
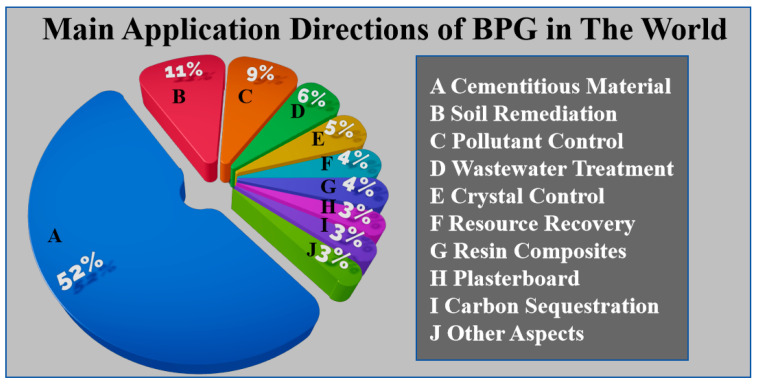
Main directions of BPG application worldwide. Note: Data collected from Web of Science; keywords: gypsum; time period: 2021–2023 [36]. This figure is a summary diagram and does not involve copyright issues.

BPG is an industrial solid waste primarily composed of calcium sulfate. Its main components include desulfurized gypsum (DG), phosphogypsum (PG), and titanium gypsum (TG), with smaller amounts of fluorogypsum, salt gypsum, and others [1,2,3]. Figure 1 shows the distribution proportions of BPG sources in China. BPG contains various impurities and harmful elements, limiting its applications [4]. Additionally, the large-scale stacking of BPG has irreversible negative impacts on the environment [5]. As of 2020, the accumulated stockpile of BPG in China exceeded 1100 million tons, making its disposal and utilization urgent tasks [6]. BPG finds widespread applications, and we compiled data on its uses over the last three years (2021–2023) from the Web of Science, as shown in Figure 2. Analysis reveals that the primary application of BPG is in construction-related cementitious materials. Therefore, we have summarized the specific applications of high-emission DG, TG, and PG in cementitious materials.

In 2020, China discharged more than 78 million tons of DG, 28 million tons of TG, and 74 million tons of PG, while their stockpiles grew to 280 million tons, 200 million tons, and 600 million tons, respectively [6]. The presence of chloride ions in desulfurized gypsum, iron, and magnesium ions in TG, as well as soluble phosphorus and fluoride in phosphogypsum, restrict their hydration performance in cementitious materials [7,8]. Moreover, heavy metal ions in BPG pose challenges to the safety of cementitious materials. However, it is noteworthy that BPG plays a crucial role in enhancing the performance of cementitious materials [9,10,11]. We analyzed the effect of BPG on the mechanical properties of cementitious materials through the Web of Science database (2021–2023). From the perspective of big data, we examined the positive impact of BPG on the development of cementitious materials’ properties. Additionally, we focused on the enhancement of the hydration activity of other industrial solid wastes in cementitious materials due to the presence of BPG. Generally, the compressive strength increases by 7–30% with the addition of BPG, data sources can be found in Section 4 (Discussion). Furthermore, under specific conditions, the mechanical properties can be improved by 80–90%. In summary, we observed that the sulfate activation of BPG has positive effects on cement (ordinary Portland cement, sulfate-resistant cement, white cement, etc.), silica–alumina solid waste (coal gangue, steel slag, blast furnace slag, etc.), and alkaline solid waste (red mud, calcium carbide slag, alkaline sludge, etc.), as illustrated in the Graphical Abstract. BPG not only enhances the hydration activity of industrial solid waste in cementitious materials but also increases the utilization rate of industrial solid waste. Moreover, with appropriate adjustments, BPG-based cementitious materials can stabilize the hazardous elements present in raw materials due to the solidification effect of ettringite [12]. The application of BPG in cementitious materials not only enables its own green value-added utilization but also promotes the recycling of other industrial solid wastes, significantly reducing material preparation costs and carbon emissions. It is worth noting that China’s cement production accounts for approximately half of the world’s total output [13,14]. In China, the cement industry is the second largest carbon emitter (steel is the first), accounting for about 10–15% of total carbon emissions [15,16]. Studies have shown that substituting one ton of raw materials with industrial solid waste in cement preparation can reduce CO_2_ emissions by 700–850 kg [17,18,19,20]. This highlights the importance of BPG in activating the joint reduction in carbon emissions across multiple industries.

From Table 1, it is evident that the research areas on the review of industrial-byproduct-gypsum-based cementitious materials primarily focus on “Products and Performance”. Additionally, PG, DG, and TG are the main gypsum-based solid wastes studied. The reviewed literature almost entirely omits articles on the hydration reaction mechanisms and harmful element stabilization mechanisms of BPG, and there is no mention of BPG’s co-utilization with other industrial solid wastes in cementitious material preparation. Unlike existing reviews, this study specifically examines the positive impacts and mechanisms of BPG on mechanical and environmental performance in cementitious materials. It also explores the synergistic patterns and mechanisms of BPG and other industrial solid wastes during the hydration process. Moreover, an environmentally friendly low-carbon cementitious material has been identified, namely, BPG + silica–alumina solid waste + alkaline solid waste based cementitious material. This review aims to provide theoretical support for researchers and offer a new non-hazardous disposal solution for environmental policymakers. The application of BPG in cementitious materials is beneficial for the joint value-added treatment of various industrial solid wastes, as illustrated in the Graphical Abstract.

**Table 1:** Research Areas in reviews on industrial byproduct gypsum.

References	Types of Gypsum	Title	Research Areas
[21]	DG	Research progress on comprehensive utilization of flue gas desulfurization gypsum and gypsum slag in smelting industry	Products and Performance
[22]	DG	A comprehensive review of flue gas desulphurized gypsum: Production, properties, and applications	Products and Performance
[23]	DG	Production and resource utilization of flue gas desulfurized gypsum in China—A review	Products and Performance
[24]	DG	Research progress and application of alpha-hemihydrate gypsum preparation from desulfurization gypsum	Hemihydrate Gypsum
[25]	PG	Research progress on phosphogypsum utilization in building materials	Products and Performance
[26]	PG	Collaborative Utilization Status of Red Mud and Phosphogypsum: A Review	Products and Performance
[27]	PG	Research hotspots and trends of comprehensive utilization of phosphogypsum: Bibliometric analysis	Products and Performance
[28]	DG and PG	Research progress on recycling of gypsum solid waste	Products and Performance
[29]	DG and PG	Preparation of α-calcium sulfate hemihydrate from industrial by-product gypsum: A review	Hemihydrate Gypsum
[30]	DG and PG	Resource utilization approach of industrial gypsum and its prospect	Products and Performance
[31]	TG	Production, characterisation, and application of titanium gypsum: A review	Products and Performance Heavy Metal Adsorption (soil)
[32]	Gypsum	Construction, renovation and demolition (CRD) wastes contaminated by gypsum residues: Characterization, treatment and valorization	Recycling and Utilization
[33]	Gypsum	Bibliometric study of the application of gypsum residues and by-products in Portland cement and mortar	Cement Preparation
[34]	Gypsum	Study on retarding feature and retardation mechanism of various retarding materials on gypsum as a construction material: A review	Retarding Feature and Retardation Mechanism
[35]	BPG	Application of the Industrial Byproduct Gypsum in Building Materials: A Review	Products and Performance

Note: Source: Web of Science; period: 2020–2024; keywords: gypsum; filter criteria: cementitious materials.

## 2. Characteristics of Industrial Byproduct Gypsum

### 2.1. Physical and Chemical Properties of BPG

Based on the above discussion, it is clear that BPG mainly consists of DG, PG, and TG, with smaller amounts of fluorogypsum, salt gypsum, citric acid gypsum, and others. It is noteworthy that DG, PG, and TG account for 88% of the total BPG in China [6]. Therefore, the large-scale utilization of these three types of solid waste is crucial for the safe disposal of BPG. This review will primarily focus on DG, TG, and PG.

This study mainly introduces three types of BPG with large stockpiles, namely, DG, TG, and PG (Table 2 shows the main chemical compositions). DG is an industrial solid waste that comes from removing SO_2_ in flue gas from coal or oil combustion with lime slurry. It is also called flue gas desulfurization gypsum [37,38]. The byproducts of this technique include calcium sulfate and calcium bisulfite (which oxidizes to calcium sulfate) and are transformed into DG. DG primarily consists of calcium sulfate dihydrate (CaSO_4_·2H_2_O) and contains calcium carbonate, calcium sulfite, calcium chloride, and small amounts of heavy metal elements (Cr, Cd, Cu, Zn, As, and Hg). CaCl_2_ has a strong corrosive effect on cementitious materials, and DG is predominantly white or pale yellow in color [39]. The local ecosystem faces a medium to high risk level from heavy metal elements in DG according to research [40,41]. For instance, the mercury in DG can leach up to 0.65 tons per year [42]. With increasing environmental awareness and regulations, the global stockpile of flue gas desulfurization gypsum is expected to grow [43].

TG is a byproduct of making titanium dioxide with the sulfuric acid method [44,45]. It is an industrial solid waste. This technique neutralizes acidic wastewater with alkaline substances such as limestone, lime, and calcium carbide slag, resulting in a precipitate primarily composed of calcium sulfate dihydrate. Additionally, impurity elements such as iron, silicon, and magnesium are present in TG, giving it colors such as green, yellow, or reddish-yellow. TG also contains small amounts of heavy metals. The presence of impurity elements such as iron and magnesium affects the hydration performance of TG in cementitious materials [46,47]. TG easily contaminates groundwater and crops and can cause dust pollution, posing health risks to humans [48,49]. As the titanium dioxide industry continues to grow, the global stockpile of TG is expected to increase.

PG is an industrial solid waste that is discharged during the wet phosphoric acid process, and it is primarily composed of calcium sulfate dihydrate [6,36,50,51]. Additionally, PG contains incompletely decomposed phosphate ore, phosphoric acid, fluoride, organic substances, and small amounts of heavy metal elements [52]. The global annual emissions of PG can reach up to 300 million tons [53]. PG comes in two colors: gray–black and gray–white. Soluble phosphoric acid and fluoride have a significant negative impact on the hydration performance of cementitious materials, limiting their large-scale utilization [54]. The strong acidity of PG can cause substantial corrosion to buildings around its storage area, posing significant threats to the safety of residents and the environment. In 2016, a collapse of a PG stockpile at Mosaic resulted in the discharge of approximately 760,000 cubic meters of contaminated water, causing significant human and environmental disasters locally. Phosphoric acid finds applications in various industries, such as agriculture, industry, food, and medicine, leading to an increasing global stockpile of phosphogypsum. The unregulated disposal of BPG seriously damages the ecosystem, polluting groundwater resources and wasting land resources.

**Table 2:** Main chemical composition of BPG [5,55,56,57,58].

Chemical Composition/%	DG	PG	TG
References	[55,56]	[5,55,57]	[55,58]
SO_3_	36.90–44.84	38.00–55.00	31.60
CaO	30.10–32.49	22.42–39.00	32.20
MgO	3.66–5.32	0.01–2.02	0.05–3.00
SiO_2_	0.7–3.30	0.37–8.62	1.90–2.90
Al_2_O_3_	0.42–1.00	0.13–0.80	0.70
Fe_2_O_3_	0.22–0.30	0.03–0.47	28.99
TiO_2_	0.04–0.05	0.03–3.57	3.40
P_2_O_5_	0.01–0.08	0.70–5.00	-
K_2_O	0.10	0.02–0.34	-
Na_2_O	0.13	0.25–0.69	-
LOl	20.18–22.40	17.00–20.00	-
Trace elements (mg/kg)			
Mn	90.00–403.00		2000–2500
Mo	0.50–14.70	0.18	0.01–7.00
Zn	2.50–14.30	15.60–250.00	170–250
Cu	0.10–7.60	0.60–2000.00	10–40
Cr	65.00–91.00	6.30–300.00	100–300
Cd	<0.01	0.25–20.00	0.90–2.00
Pb	3.00–218.00	0.01–400.00	10–40
Ni	1.50–17.30	3.50–150.00	20–40
F		0.20–1.00 (%)	
As	<2.60	0.06–25.00	12–150
Se	<2.10	0.14–30.00	
Cl	0.05–0.40 (%)		
Hg	0.25	0.03–8.00	0.01–271
B	98.00–175.00		
Co		0.60–20.00	10.00–20.00
Sr		350.00–370.00	
Ba		36.00–38.00	
Eu			0.52 (%)
Ru			0.80 (%)
V			0.44 (%)

### 2.2. The Role of BPG in Cementitious Materials

With in-depth research on BPG, scholars have increasingly recognized its significant advantages when utilized in the preparation of cementitious materials [8,59]. BPG plays a crucial role in improving the mechanical properties of cementitious materials [60]. This is because BPG accelerates the rate and yield of hydration products (such as ettringite and C-S-H) during the hydration process [61,62]. The needle-like structure of ettringite fills the pores between cementitious products, enhancing the matrix’s density and stability. The intertwined growth of ettringite and C-S-H (mainly consisting of gel products containing Ca, Si, and H2O) forms a stable network structure within the matrix. The larger specific surface area of BPG allows it to have more extensive contact with other raw materials, promoting the development of the hydration reaction surface area and enhancing the formation of the hydration product network structure. Gypsum particles that do not participate in chemical reactions are gradually enveloped by an evolving network structure, reducing their solubility and enhancing the matrix’s water resistance and corrosion resistance [61]. The pore structure of the matrix is also optimized by these unreacted gypsum particles, which can act as microaggregates [63,64]. Moreover, BPG exhibits a certain retarding effect in cementitious materials, improving their flowability and workability. This is because BPG reduces the yield stress and viscosity of cementitious materials. Additionally, an appropriate amount of BPG can compensate for the reduction in volume of cement-based cementitious materials due to the volume change in ettringite [65]. Gypsum is usually classified into hemihydrate (CaSO_4_·0.5H_2_O) and dihydrate (CaSO_4_·2H_2_O), with hemihydrate being further divided into α-hemihydrate (α-HH) and β-hemihydrate (β-HH). α-HH is obtained through dissolution and recrystallization in solution, while β-HH can be obtained through high-temperature evaporation [66]. Hemihydrate has attracted more and more attention because it is beneficial to the development of cementing material properties. Under specific conditions, hemihydrate is superior to dihydrate in enhancing the hydration performance of cementitious materials [67,68]. Simultaneously, BPG, due to its unique sulfate activation effect, significantly enhances the hydration activity of cement and other industrial solid wastes (alkaline solid waste and silica–aluminous solid waste). It is noteworthy that gypsum-based cementitious materials also play an important role in the stable solidification of hazardous elements in raw materials [69]. Gypsum accelerates the rate and yield of ettringite formation during hydration. Ettringite’s unique chemical structure has a good solidification effect on heavy metal oxyanions and cations [70,71]. Additionally, products such as C-S-H formed during the hydration reaction also have a positive impact on the solidification of hazardous elements [72]. The influence of BPG on the hydration characteristics and mechanisms of cementitious materials, as discussed in Section 2.2, will be discussed in depth in Section 3.

## 3. The Influence of Industrial Byproduct Gypsum on the Properties of Cementitious Materials

### 3.1. Mechanical Properties

#### 3.1.1. Desulfurized Gypsum

The performance parameters of the BPG-based cementitious materials involved in the third part are shown in Table 3.

Lv et al. [73] investigated the influence of desulfurized gypsum (DG) on the rheological properties and expansion rate of ordinary Portland cement (OPC). The study revealed that the impact of DG on the rheological behavior of OPC slurries approximates that of a Newtonian fluid. Additionally, DG significantly affects the shear stress of the OPC slurry. As the DG content increases, the viscosity of the OPC slurry gradually decreases. The linear relationship between shear stress and shear strain in the slurry conforms to the Bingham rheological equation. This is because DG exhibits a certain retarding effect. When the DG content in the OPC slurry is increased, the yield stress and viscosity of the slurry decrease, improving its overall flowability and ensuring good workability. During the hydration process of OPC, the volume is reduced, whereas specimens containing DG expand. Moreover, with an increase in DG content, the expansion rate of the specimens gradually increases. This is because the addition of DG generates ettringite in the DG–OPC matrix, and its content is directly proportional to the amount of DG added. Ettringite increases the volume of DG–OPC. It is worth noting that DG can counteract the reduction in OPC volume during hydration, which prolongs the service life of DG–OPC.

Calcium sulfoaluminate cement (CSA) is a cementitious material formed through the low-temperature calcination of raw materials such as limestone and gypsum, and it consists mainly of anhydrous calcium sulfoaluminate (C_4_A_3_S) and dicalcium silicate (C_2_S) minerals [74]. It possesses properties such as low carbon emissions, low alkalinity, and high strength. However, with the rising cost of gypsum minerals, the production cost of CSA has also increased. To reduce the cost of CSA, Xu et al. [75] studied the influence of DG on the performance and microstructure of CSA. The study revealed that the curing temperature of DG and DG–CSA is a crucial factor in the hydration process of CSA. DG facilitates the formation of more ettringite and aluminum hydroxide gel, enhancing the mechanical properties of DG–CSA during the hydration process. At a curing temperature of 40 °C, DG can also inhibit the formation of monosulfate and strätlingite during hydration, positively influencing the volume stability and mechanical properties of DG–CSA. This is because monosulfate and ettringite have different densities; excessive monosulfate can significantly alter the volume of DG–CSA, thereby deteriorating its mechanical properties. The main chemical reactions occurring during the hydration process of DG–CSA are as follows:

(1) $$C_{4}A_{3}\bar{S}+2C\bar{S}H_{2}+34H\to C_{6}A{\bar{S}}_{3}H_{32}+2AH_{3}\;(\mathrm{gel})$$

(2) $$C_{4}A_{3}\bar{S}+6\mathrm{CH}+8C\bar{S}H_{2}+74H\to 3C_{6}A{\bar{S}}_{3}H_{32}$$

(3) $$C_{4}A_{3}\bar{S}+18H\to C_{4}A\bar{S}H_{12}+2AH_{3}\;\left(\mathrm{gel}\right)$$

(4) $$6\mathrm{CH}+2AH_{3}+C_{6}A{\bar{S}}_{3}H_{32}\to 3C_{4}A\bar{S}H_{12}+8H$$

A ternary cementitious system (MgO–MgSO_4_–H_2_O) consisting of MgO and MgSO_4_ solution is called magnesium oxysulfate cement (MOSC) [76]. However, its widespread application is limited due to its high cost and volume instability. Gu et al. [77] modified MOSC with DG to reduce its cost while enhancing its performance. The study revealed that DG–MOSC demonstrates good mechanical properties, water resistance, and volume stability. Unlike in MOSC systems, the presence of DG promotes the formation of an acicular 5Mg(OH)_2_·MgSO_4_·7H_2_O (5·1·7) phase in DG–MOSC systems. The 5·1·7 phase exhibits excellent strength, thereby enhancing the mechanical properties of DG–MOSC. Additionally, the addition of DG transforms the poorly volume-stable Mg(OH)_2_ gel phase into the 5·1·7 phase, which possesses superior volume stability. The corresponding chemical reaction equations are as follows:

(5) $$\mathrm{MgO}+(x+1)H_{2}O\to [\mathrm{Mg}(\mathrm{OH})(H_{2}O{)}_{x}{]}^{+}+OH^{-}$$

(6) $$\begin{array}{l} 2[\mathrm{Mg}(\mathrm{OH})(H_{2}O{)}_{x}{]}^{+}+SO_{4}^{2-}+4Mg^{2+}+8OH^{-}\to \\ 5\mathrm{Mg}(\mathrm{OH}{)}_{2}\cdot {\mathrm{MgSO}}_{4}\cdot 7H_{2}O+(2x-7)H_{2}O \end{array}$$

Blast furnace slag (BFS) is a silico–aluminous solid waste discharged during the iron smelting process in blast furnaces, and it possesses potential cementitious activity [78]. BFS is often used in combination with cement to prepare cementitious materials. Increasing evidence indicates that the appropriate addition of DG can better activate its latent pozzolanic activity. The effect of DG on the characteristics of cementitious materials based on BFS was investigated by Shi et al. [79]. The study revealed that the addition of DG significantly enhances the performance of BFS-based cementitious materials. When the SO_3_ content is within 3.5%, the mechanical properties of the matrix increase proportionally with the increase in DG. Additionally, adding DG after calcination at 200 °C results in an optimal 28-day compressive matrix strength, reaching 53 MPa. This enhancement is attributed to the high content of Ca^2+^ and SO_4_^2−^ in DG, which react with dissolved SiO_4_^4−^ and AlO_4_^4−^ in BFS, producing hydrated calcium silicate (C-S-H) and ettringite and leading to a denser microstructure within the matrix. Moreover, alkali metal salt minerals present in DG (potassium and sodium salts) accelerate the hydration reaction of cement, stimulating the matrix’s activity. Under high-temperature conditions, changes in the crystal structure of DG positively influence the activation of BFS-based cementitious materials. Furthermore, the large specific surface area of DG ensures increased contact with raw materials, expediting the hydration reaction.

This study suggests that adding BPG separately to OPC can lead to the erosion of the matrix structure by sulfates. This is because BPG can react with calcium aluminate hydrates (e.g., 4CaO·Al_2_O_3_·13H_2_O) or monosulfoaluminate hydrate (3CaO·Al_2_O_3_·CaSO_4_·12H_2_O) to generate delayed ettringite (3CaO·Al_2_O_3_·3CaSO_4_·32H_2_O). The reaction equation is as follows:

(7) $$C_{4}AH_{x}+2Ca^{2+}+3SO_{4}^{2-}+(32-x)H_{2}O\to 3\mathrm{CaO}\cdot Al_{2}O_{3}\cdot 3\mathrm{CaS}O_{4}\cdot 32H_{2}O$$

(8) $$\begin{array}{l} 3\mathrm{CaO}\cdot Al_{2}O_{3}\cdot \mathrm{CaS}O_{4}\cdot xH_{2}O+2Ca^{2+}+2SO_{4}^{2-}+(32-x)H_{2}O\to \\ 3\mathrm{CaO}\cdot Al_{2}O_{3}\cdot 3\mathrm{CaS}O_{4}\cdot 32H_{2}O \end{array}$$

BPG and OPC may also produce a more dangerous compound called thaumasite (CaSiO_3_·CaCO_3_·CaSO_4_·15H_2_O). The relevant chemical reaction equations are as follows:

(9) $$\begin{array}{l} \mathrm{Ca}(\mathrm{OH}{)}_{2}/3\mathrm{CaO}\cdot 2{\mathrm{SiO}}_{2}\cdot 8H_{2}O+SO_{4}^{2-}+CO_{3}^{2-}+H_{2}O\to \\ {\mathrm{CaSiO}}_{3}\cdot {\mathrm{CaCO}}_{3}\cdot {\mathrm{CaCO}}_{3}\cdot {\mathrm{CaSO}}_{4}\cdot 15H_{2}O \end{array}$$

Meanwhile, adding a large amount of DG separately to OPC amplifies its defects, leading to low compressive strength and slight water solubility. Dehydrated DG will reform gypsum crystals during the hydration process. The relevant reaction equations are as follows:

(10) $$\mathrm{CaS}O_{4}\cdot 2H_{2}O\overset{120–180{}^{\circ}C}{\to }\mathrm{CaS}O_{4}\cdot 0.5H_{2}O+1.5H_{2}O$$

(11) $$\mathrm{CaS}O_{4}\cdot 0.5H_{2}O+XH_{2}O\to \mathrm{CaS}O_{4}\cdot 2H_{2}O+(X-1.5)H_{2}O$$

It is worth noting, that if the formation of ettringite can be controlled in the early stages of hydration, the abovementioned risks can be reduced [80]. To address these risks, Wansom et al. [61] simultaneously added DG and fly ash (FA) to OPC in appropriate proportions. The cementitious DG–FA–OPC system not only mitigates the risk of sulfate erosion and the high solubility of DG in water, but also enhances the mechanical properties and water resistance of the matrix. Studies indicate that FA can accelerate sulfate consumption in the early hydration stages, promoting the formation of early ettringite (a major reason for early strength development) and preventing the formation of delayed ettringite. The reduction in OPC content in the system leads to a decrease in Ca(OH)_2_ content. Additionally, the SiO_2_ in FA undergoes a pozzolanic reaction with Ca(OH)_2_. The lower amount of Ca(OH)_2_ prevents the formation of thaumasite. The hydration process produces less monosulfoaluminate hydrate, which can form delayed ettringite with gypsum, because of the lower amount of 3CaO·Al_2_O_3_ (from OPC). Moreover, C-S-H and ettringite encapsulating gypsum particles can reduce the solubility of gypsum in water, enhancing the matrix’s water resistance. It is noteworthy that this study provides a direction for the high-dosage application of DG, namely, increasing the dosage of DG by adding raw materials with pozzolanic activity to reduce the OPC content.

**Figure 3:**
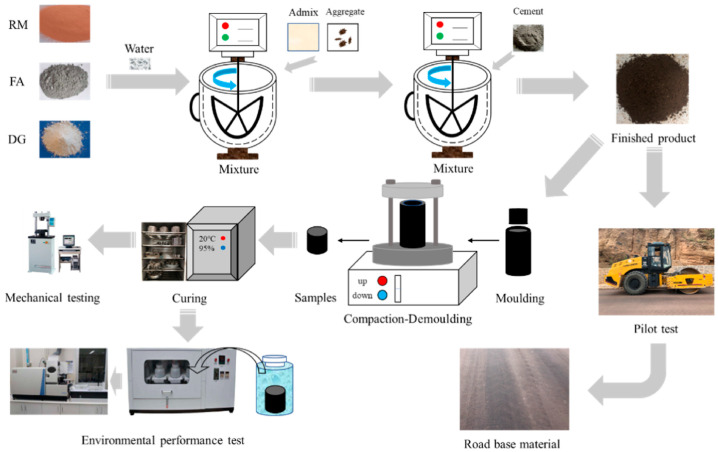
Preparation process of DG–RM–FA–OPC-based road base materials [81].

Li et al. [81] investigated the influence of DG on a red mud (RM, alkaline solid waste)–FA (silico–aluminous solid waste)–OPC binder system. The study revealed that DG had a positive impact on the enhancement of the RM–FA–OPC system’s performance. When (CaO + Na_2_O)/(SiO_2_ + Al_2_O_3_) = 0.88, the 7-day compressive strength of the DG–RM–FA–OPC meets the requirements for highways. The preparation process of the DG–RM–FA–OPC-based road base material is illustrated in Figure 3. C-A-S-H gel, ettringite, and zeolite are the main hydration products of DG–RM–FA–OPC. They improve the road base material’s strength and durability. DG–RM–FA–OPC has a compressive strength that is 1.6 times higher than that of RM–FA–OPC, which is remarkable. The addition of DG effectively activated the synergistic effects among the raw materials in the system. Ca^2+^ and SO_4_^2−^ indirectly increased the alkaline activation degree of RM and the leaching content of active silico–aluminate in FA. Simultaneously, the substantial formation of ettringite optimized the pore structure of the matrix. Ettringite and the existing gel phases in the system grew together and intertwined, facilitating the development of the system’s mechanical properties. The relevant reaction equations are as follows:

(12) $$\mathrm{Si}O_{2}+OH^{-}+H_{2}O\to [H_{3}\mathrm{Si}O_{4}{]}^{\_}$$

(13) $$\mathrm{Al}O_{2}+OH^{-}+H_{2}O\to [H_{3}\mathrm{Al}O_{4}{]}^{2-}$$

(14) $$[H_{3}\mathrm{Si}O_{4}{]}^{-}+[H_{3}\mathrm{Al}O_{4}{]}^{2-}+Ca^{2+}\to C-A-S-H$$

(15) $$\mathrm{Al}O_{2}^{-}+OH^{-}+H_{2}O\to [\mathrm{Al}(\mathrm{OH}{)}_{6}{]}^{3-}$$

(16) $$2[\mathrm{Al}(\mathrm{OH}{)}_{6}{]}^{3-}+6Ca^{2+}+3SO_{4}^{2-}+26H_{2}O=Ca_{6}Al_{2}(SO_{4}{)}_{3}(\mathrm{OH}{)}_{12}\cdot 26H_{2}O(\mathrm{AFt})$$

AFm (monosulfate phase) formed between 90 and 180 days of hydration, which reduced the system’s strength. The formation of AFm occurred due to the conversion of tetrahedral aluminum in the system into octahedral aluminum, which then reacted with Ca^2+^ and SO_4_^2−^.

**Figure 4:**
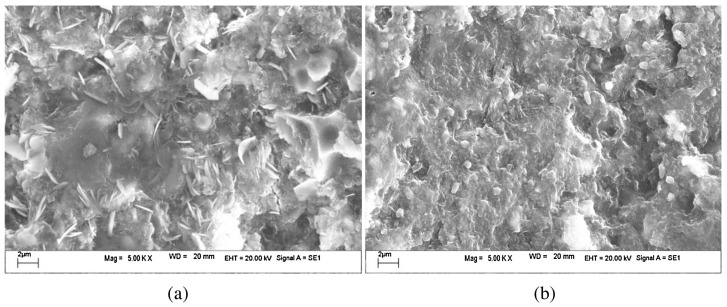
Changes in the main hydration products in the matrix from 3 d to 90 d under the activation of DG, (**a**) 3 d; (**b**) 90 d [83].

Calcium silicate slag (CCS), a caustic byproduct of alumina extraction from fly ash, is a solid waste with high alkalinity and corrosivity [82]. However, it contains calcium, silicon, and aluminum resources, making it a potential raw material with cementitious activity. Zhang et al. [83] studied the activating effect of DG as an additive on a CCS (alkaline solid waste)-based cementitious system. This system also includes some silico–aluminate solid wastes (BFS and FA) and OPC. The study showed that the main hydration products are C-A-S-H (amorphous), ettringite (rod-like), and Ca(OH)_2_ (hexagonal plates). These products compact the matrix and enhance its mechanical properties. The addition of DG increased the rate and extent of transformation of active silico–aluminate minerals into ettringite and gel phases in the system. Simultaneously, DG indirectly accelerated the leaching rate of active SiO_2_ and AlO^2−^ from CCS, BFS, and FA, accelerating the hydration reaction of the matrix. The changes in the main hydration products in the matrix from 3 days to 90 days can be clearly seen in Figure 4. The amorphous C-A-S-H gradually increased and became thicker and denser. Meanwhile, the hexagonal plate-like Ca(OH)_2_ gradually decreased, which was due to DG accelerating the pozzolanic reaction between CCS, BFS, FA, and Ca(OH)_2_.

At the current technological level, the cost of producing α-HH using BPG is relatively high, which hampers the large-scale application of BPG in cementitious materials. Increasing the cost-effectiveness of preparing β-HH using BPG has gradually become a research focus [84]. Hao et al. [84] utilized CaO as a crystal modifier to calcine DG, enhancing the quality of β-HH. During the calcination process, DG transformed from monoclinic DG to monoclinic β-HH. The addition of CaO transformed the calcination product into orthorhombic β-HH. DG lost 1.5 crystal water molecules in the absence of CaO, and CaO changed the crystal shape. Its Ca^2+^ ions promoted the growth of hemihydrate gypsum’s (400) face and inhibited the growth of units on the (200) face. The mechanical properties of materials are improved by β-HH. Moreover, as the β-HH content increases, the setting time of DG-based cementitious materials drops. The setting time mainly depends on the quick dissolution of β-HH and the formation of hydration products. Therefore, the more β-HH there is, the faster the material hydrates, leading to a shorter setting time [85].

The main impurity in DG is CaCl_2_. Even a small amount of CaCl_2_ in cementitious materials for construction can cause severe corrosion to the matrix. To address this issue, Li et al. [86] used BFS and white calcium aluminate cement (CAC) to optimize the environmental and mechanical performance of DG-based cementitious materials. This study found that the addition of CAC and BFS improves the hydration reaction and the water resistance of β-HH. Compared with DG-based cementitious materials, the compressive strength and water resistance of the DG(β-HH)–BFS–CAC system increased by 59.8% and 61.5%, respectively. Through their synergistic effects, CaO–Al_2_O_3_ (CA) rapidly reacts with β-HH to form AFt or AFm. At the same time, CaCl_2_ in the system forms Friedel’s salt (Ca_4_Al_2_(OH)_12_Cl_2_·4H_2_O) and CaCl_2_·4H_2_O. It also participates in the hydration reaction and fills or encapsulates the pores between ettringite and the gel products, making the structure denser. Additionally, the complex network structure of the hydration products wraps around unreacted gypsum and prevents its dissolution.

**Table 3:** Properties of BPG-based cementitious materials.

System	Hydration Conditions		Compressive Strength (MPa, 28 d)	Other Properties	References
	Usage	T/°C			
DG–CSA	DG	40	39.0–8% Increase Compared to CSA	Drying shrinkage DG–CSA: 0.08~0.15% CSA: 0.00~0.28%	[75]
	10%				
DG–MOSC	SO_4_^2−^	RT	81.1–11% Increase Compared to MOSC	Setting time DG–MOSC: 280–450 min MOSC: 230–380 min	[77]
	3.54 g·L^−1^				
				Drying shrinkage DG–MOSC: 0–0.25% MOSC: 0–0.49%	
DG–BFS–OPC	SO_3_	200	53.0		[79]
	3.5%				
DG–FA– OPC	DG	RT	38.57	Initial setting time > 45 min	[61]
	20%				
DG–RM– FA–OPC	DG	RT	10.99–214% Increase Compared to RM–FA–OPC	Expansion rates DG–RM–FA–OPC: 0.01% RM–FA–OPC: 0.02%	[81]
				freeze resistance (Compressive Strength Loss Rate) DG–RM–FA–OPC:0–10% RM–FA–OPC: 10–20%	
	5%			Heavy metal ion solidification rate Na: 75% As: 97% Hg: 91% Zn: 99% Pb: 99% Cu: 53%	
DG–CCS–BFS–FA–OPC	DG	RT	37.5	Setting time 95–160 min	[83]
	6%			Leachate results/ppm AS: <0.001 Cr: <0.001 Pb: <0.001 Cu < 0.182 Cr < 0.001 Cd < 0.001	
TG–FA– CCS	m(TG/ FA)	RT	18.93–3% Increase	Heavy metal ion solidification rate Cu: >99% Mn: >99% Cr: >48%	[47]
	0.23				
TG–HFA– CCS	TG	RT	22.02–55% Increase Compared to HFA– CCS	Initial setting time TG–HFA–CCS: 800 min HFA–CCS: 850 min	[46]
	12.5%				
TG–RM–OPC	TG	RT	11.8–58% Increase Compared to RM–OPC	PH TG–RM–OPC: 11.2 RM–OPC: 12.5	[87]
	10%				
TG–TS–OPC	TG	RT	45–27% Increase	Softening coefficient: 0.74	[88]
	30%			Drying shrinkage: 0.15%	
TG–RBRS–OPC	TG	115	4.8–87.5% Increase Compared to RBRS–OPC		[89]
	3%				
TG–BFS–DG	TG	RT	27–2% Increase Compared to BFS–DG	setting time TG–BFS–DG: 23–89 min BFS–DG: 25–87 min	[62]
	30%				
TG–SAC–OPC	TG	RT	17.9–49% Increase	Fluidity: 118 mm	[90]
	45%			Shrinkage: 0.135%	
TG–RM–OPC	TG	RT	8–166% Increase Compared to RM–OPC	Concentration of heavy metal ions in leachate meets GB 18582-2008	[60]
	10%				
DG (β-HH)	DG	RT	10.6 (3 d)	Meet the requirements of 3.0 grade building gypsum	[84]
	100%			setting time: 4.2–6.8 min	
DG–BFS–CAC (β-HH)	DG	RT	13.9–59.8% Increase Compared to DG	setting time: 9–25 min	[86]
	100%			Softening coefficient 0.42	
				Chloride binding (%) DG–BFS–CAC: 38.4 DG: 0	
PG–yellow clay–NaOH	PG	RT	27–13% Increase Compared to yellow clay–NaOH	water absorption PG–yellow clay–NaOH: 1.5% yellow clay–NaOH: 3%	[91]
	20%				
PG–FA–SS	PG	RT	28–18% Increase Compared to PG–FA	Setting time PG–FA–SS: 520–575 min PG–FA: 405–480 min	[92]
	15%				
PG	PG	RT	37.6 (3 d)	Concentration of heavy metal ions in leachate meets GB 18582-2008	[93]
	100%				
PG–FA–SS	PG	RT	8.36	resilience modulus: 1987 MPa	[94]
	2.4%			splitting strength reaches: 0.82 MPa	
PG–OPC	SO_3_	RT	34		[95]
	4.0%				
PG–OPC	PG	RT	44.7	The hydration acceleration period is advanced 17–21 h	[96]
	25%				
PG–CCS– CFBFA	PG	RT	11.4–33% Increase Compared to CCS– CFBFA (14 d)		[97]
	40%				
PG–CSA	PG	RT	88–487% Increase Compared to CSA	Setting time PG–CSA: 3–70 min CSA: 125–230 min	[98]
	20%				
PG (α-HH)	PG	RT	52.4 (2 h)–45%	Setting time: 14–52 min	[99]
	100%			Normal consistency: 0.68 to 0.31	

Note: RT = room temperature.

#### 3.1.2. Titanium Gypsum

Red-bed weathered residual soil (RBRS) possesses characteristics such as a high solubility upon contact with water and a soft rock structure. When used as a road base material, RBRS can promote issues such as road surface collapse. Huang et al. [89] modified RBRS using a combination of TG and OPC to prepare TG–OPC-based cementitious materials. The study revealed that the compressive strength of the stabilized soil increased significantly from 0.5 MPa to 4.8 MPa after modification for 28 days. The addition of TG–OPC resulted in a denser internal structure of RBRS and a noticeable reduction in porosity. The synergy between TG–OPC and RBRS was evident. OPC hydrolysis increased the ion concentration in the soil electrolyte by producing Ca^2+^ and OH^−^. Ca^2+^ ions replaced Na^+^ and K^+^ on the RBRS surface. TG’s sulfate environment sped up the ion exchange rate [100]. This process changed the surface charges of the RBRS, thinning the electric double layer between soil particles and decreasing the RBRS swelling potential [101]. The ion exchange reactions involving TG in the soil are as follows:

(17) $$\mathrm{CaS}O_{4}\cdot 0.5H_{2}O+H_{2}O\overset{Dissolving}{\to }Ca^{2+}+SO_{4}^{2-}+1.5H_{2}O$$

(18) $$K^{+}(s)+Na^{+}(s)+Ca^{2+}(l)\overset{\mathrm{Ionsexchange}}{↔}Ca^{2+}(s)+Na^{+}(l)+K^{+}(l)$$

**Figure 5:**
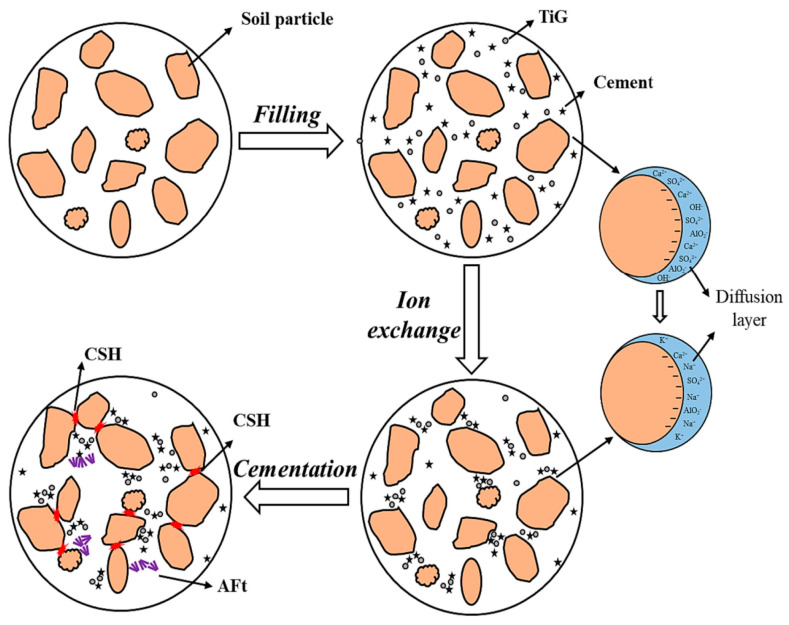
Mechanism of TG−OPC−based cementitious materials improving RBRS [89].

Simultaneously, the surfaces of the TG particles contain a significant number of free and unsaturated bonds. In water, TG reacts with H^+^ and OH^−^, forming many silica hydroxyl groups. These groups speed up cement hydration. The bonding strength between soil particles and soil density are improved by the increase in hydration products (such as ettringite and C-S-H), creating a three-dimensional network structure. The mechanism by which TG–OPC-based cementitious materials improve RBRS is illustrated in Figure 5 below.

FA is a powdery silico–aluminate solid waste collected after coal combustion [102,103]. the FA accumulation has greatly increased with the development of the power industry, posing a serious challenge to environmental safety. To improve the utilization rate of FA, Chen et al. [47] modified FA with TG and optimized the matrix’s performance through a response surface model. The study showed that the matrix’s compressive strength rose by 41% in 28 days when the TG/FA mass ratio went from 0.15 to 0.23, suggesting that TG enhanced the matrix’s mechanical properties. Fe^3+^ in TG hindered the system’s early strength growth. However, as hydration time progressed, the remaining Fe^3+^ in the matrix joined the ettringite formation, boosting the strength at prolonged timescales. This is due to the higher content of Al compared to Fe in the matrix and Al’s higher tendency to bond with ettringite. At the same time, TG’s SO_4_^2−^ combined with the matrix’s free Ca^2+^ and [AlO_4_] to produce rod-shaped ettringite, which filled the matrix’s pores and increased its compactness. The dense matrix significantly enhanced the mechanical properties of the cementitious material.

**Figure 6:**
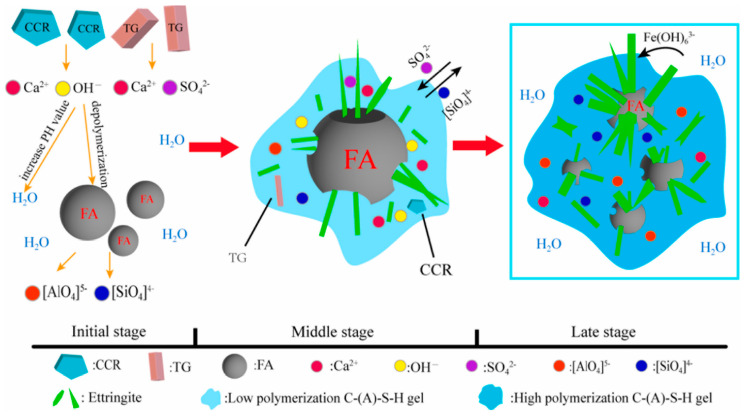
Synergistic reaction mechanism among TG, CCS, and HFA [46].

Yuan et al. [46] studied how TG affected cementitious systems based on high-calcium fly ash (HFA, a silico–aluminous solid waste) and the conversion of iron compounds from TG in cementitious materials. They found that TG raised the Ca^2+^ level in the matrix, which slowed down the Ca(OH)_2_ dissolution and the matrix’s early strength development, as shown by the equation below. Moreover, in the early hydration stage, TG introduced a considerable amount of amorphous Fe(OH)_3_ into the system, which did not participate in the early hydration reactions, impeding their reaction rate. This is also the reason why TG has a retarding effect on cementitious systems. The mechanical properties of the matrix improved significantly because of TG, despite the adverse effects of its impurities. The strength of the TG-based cementitious system at 7 and 28 days was 2.2 times and 2.6 times that of the system without TG, respectively. The small crystal structure of TG also facilitates its complete interaction and reaction with other raw materials. The alkaline activation effect of CCS and the sulfate activation effect of TG accelerates the dissolution of active silico–aluminate minerals in HFA, accelerating the hydration reaction of the matrix. In the later stages of hydration, the increase in [Si(Al)O_4_] content in the matrix indicates an increase in hydration products. As the water content in the system decreases, ion reactions transform into solid-phase reactions. Additionally, the unreacted Fe(OH)_3_ in TG transforms into Fe(OH)_6_^3−^ and directly participates in ion exchange, generating ettringite crystals (Ca_6_[A_l1_-xFe_x_(OH)_6_]2(SO_4_)_3_·26H_2_O). The displaced Al^3+^ then continues to react with free mineral phases, producing other hydration products and ettringite, further deepening the degree of hydration and compactness of the matrix. This leads to an improvement in the later-stage strength of the matrix. The reaction mechanism is illustrated in Figure 6.

(19) $$\mathrm{Ca}(\mathrm{OH}{{)}_{2_{\left(S\right)}}}_{\leftarrow }^{\to }C{a^{2+}}_{\left(aq\right)}+2O{H^{-}}_{\left(aq\right)}$$

RM is a hazardous alkaline solid waste discharged during the production of alumina [104]. The challenge in utilizing RM lies in its high alkalinity and complex composition, which lead to an increase in its accumulation year-on-year. TG is an acidic sulfate waste. Co-utilizing the characteristics of TG and RM could alleviate the difficulty of utilizing RM. Li et al. [87] modified RM with TG to create cementitious materials for road base construction. The study indicates that the prepared road base material meets the requirements for highways and primary roads. RM provides abundant OH^−^ to the TG–RM–OPC system, while TG supplies free Ca^2+^ and SO_4_^2−^. This combination creates alkaline activation and sulfate activation conditions for the active substances SiO_2_ and Al_2_O_3_ in the system, forming a network structure primarily composed of ettringite and C-S-H. This enhances the mechanical properties of the matrix. Additionally, the pH of the TG–RM–OPC system decreases with the curing time. The Ca^2+^ provided by TG undergoes displacement reactions with Na^+^ ions adsorbed on the surface of RM, promoting Na^+^ leaching. The 28-day test results show a 12% decrease in pH and a 133% improvement in mechanical performance, indicating the significant effectiveness of TG in modifying RM-based cementitious materials.

Titanium slag, a silico–aluminate industrial waste, is discharged during the smelting of pig iron using vanadium–titanium–iron ore as a raw material [105]. Its low reactivity is attributed to the presence of inert calcium titanium minerals, making it challenging to utilize. However, titanium slag contains a significant number of glassy phases, endowing it with potential cementitious activity. Jiang et al. [88] prepared TG–titanium slag (TS)–OPC-based cementitious materials with appropriate proportions using TG as an activator and sodium silicate (SSC) as an additive. This study revealed that appropriate amounts of TG under alkaline activation conditions can enhance the mechanical properties of the matrix. SSC enhances TG’s sulfate activation effect in the system. The alkaline environment accelerates the rate of reaction of Ca^2+^ and SO_4_^2−^ with the glassy phases (active silico–aluminate materials) in TS, leading to the formation of ettringite and gel phases. Furthermore, the dense network structure in the system, composed of C-S(A)-H and AFt, physically encapsulates unreacted calcium sulfate from TG, preventing its leaching and improving the softening coefficient (water resistance) of TG–TS–OPC, which increased by 57%. Additionally, the addition of TG reduced the SSC content in the system, mitigating the shrinkage of TG–TS–OPC. This is because an excess of Na^+^ can alter the stacking order between the layers of C-S(A)-H, making the material more susceptible to collapse and redistribution under drying conditions. Moreover, numerous alkali-induced chemical reactions increase the system’s autogenous shrinkage rate.

**Figure 7:**
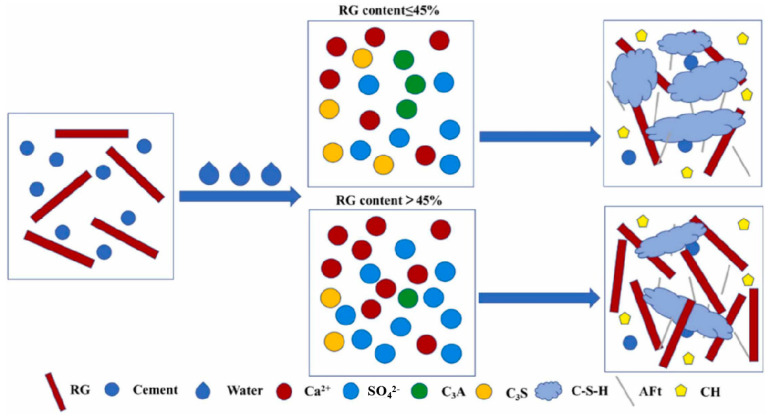
Mechanism of action of TG−SAC−OPC [90].

Wang et al. [90] used TG to modify sulfate aluminate cement (SAC)–OPC-based cementitious material and prepared self-leveling mortars. Their results showed that TG significantly enhanced the mechanical properties of the matrix. An appropriate amount of TG could compensate for the matrix shrinkage, maintaining its stable structure. TG reacted with C_3_A in the cement to form ettringite and promoted the generation of more C-S-H gel phases in the matrix. Additionally, unreacted TG served as a microaggregate, thus increasing the density. The mechanism of action for TG–SAC–OPC is illustrated in Figure 7.

#### 3.1.3. Phosphogypsum

**Figure 8:**
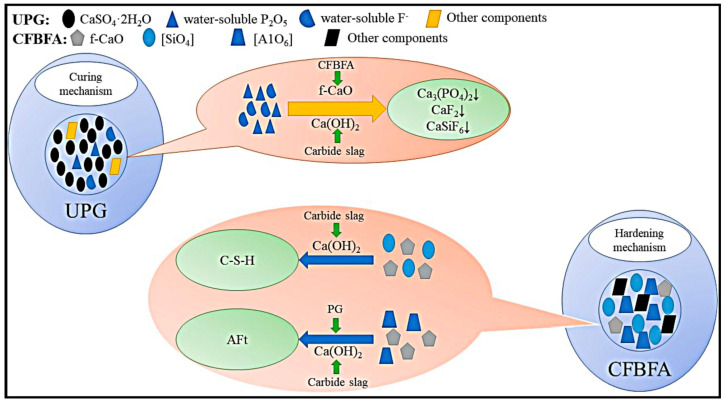
Synergistic mechanisms among CCS, CFBFA, and PG [97]. Note: UPG denotes undisturbed phosphogypsum; CFBFA denotes CFB fly ash.

Calcium carbide slag (CCS), an alkaline industrial solid waste, is generated during the hydrolysis of calcium carbide (CaC_2_) to produce acetylene gas. The extensive accumulation of calcium carbide slag has led to environmental degradation. Li et al. [97] modified CCS-based cementitious materials using phosphogypsum (PG) to achieve large-scale utilization of both CCS and PG. The system also contains CFBFA and OPC, which are types of fly ash and cement. The study reveals the excellent synergistic effects among PG, CCS, and CFBFA. PG provides abundant SO_4_^2−^, enhancing the sulfate activation effect on potential silico–aluminous minerals in CFBFA. CCS creates an alkaline environment in the system, accelerating the disaggregation of [SiO_4_] and [AlO_6_] and, thus, strengthening the hydration reaction between PG and CFBFA. CCS and CFBFA, which are modified materials, enhance PG-based cementitious materials’ performance and lower PG’s soluble phosphorus and fluoride levels. The synergistic effects among these three components lead to the formation of more C-S-H and ettringite hydrates, enhancing the mechanical properties of the matrix. Additionally, the soluble acids and fluorides in PG react with CCS and CFBFA to form inert insoluble salts (Ca_3_(PO_4_)_2_, CaSiF_6_, and CaF_2_), mitigating the negative impacts of harmful elements on the hydration reaction. The synergistic mechanisms among CCS, CFBFA, and PG are illustrated in Figure 8. The specific reactions are outlined below.

(1) Neutralization curing reaction equation

(20) $$2Na_{3}PO_{4}+3\mathrm{Ca}(\mathrm{OH}{)}_{2}=Ca_{3}(PO_{4}{)}_{2}↓+6\mathrm{NaOH}$$

(21) $$2Na_{2}\mathrm{HP}O_{4}+3\mathrm{Ca}(\mathrm{OH}{)}_{2}=Ca_{3}(PO_{4}{)}_{2}↓+4\mathrm{NaOH}+2H_{2}O$$

(22) $$2\mathrm{Na}H_{2}PO_{4}+3\mathrm{Ca}(\mathrm{OH}{)}_{2}=Ca_{3}(PO_{4}{)}_{2}↓+2\mathrm{NaOH}+4H_{2}O$$

(23) $$2H_{3}PO_{4}+3\mathrm{Ca}(\mathrm{OH}{)}_{2}=Ca_{3}(PO_{4}{)}_{2}↓+6H_{2}O$$

(24) $$Na_{2}\mathrm{Si}F_{6}+\mathrm{Ca}(\mathrm{OH}{)}_{2}=\mathrm{CaSi}F_{6}↓+2\mathrm{NaOH}$$

(25) $$2\mathrm{NaF}+\mathrm{Ca}(\mathrm{OH}{)}_{2}=\mathrm{Ca}F_{2}↓+2\mathrm{NaOH}$$

(2) Hydration process

(26) $$\mathrm{Si}O_{2}+\mathrm{xCa}(\mathrm{OH}{)}_{2}+nH_{2}O=\mathrm{xCaO}\cdot \mathrm{Si}O_{2}\cdot (n+x)H_{2}O$$

(3) The formation of ettringite

(27) $$Al_{2}O_{3}+3\mathrm{Ca}(\mathrm{OH}{)}_{2}+3\mathrm{CaS}O_{4}\cdot 2H_{2}O+27H_{2}O=3\mathrm{CaO}\cdot Al_{2}O_{3}\cdot 3\mathrm{CaS}O_{4}\cdot 32H_{2}O$$

Steel slag (SS), an alumino-silicate industrial solid waste, contains C_2_S (Ca_2_SiO_4_) and C_3_S (Ca_3_SiO_4_) with certain potential cementitious properties. However, the presence of f-CaO limits the utilization of SS [106,107,108]. f-CaO disrupts the stability of cementitious materials. To address this issue, Zhao et al. [92] modified SS-based cementitious materials using PG and prepared road base materials. The study demonstrates that an appropriate amount of PG can activate inert silico–aluminous minerals in the matrix. Moreover, it effectively suppresses the transformation of AFt into AFm, compensating for the matrix’s shrinkage effect and improving stability. As the PG content increases, the hydration induction period extends. This is because calcium phosphate and calcium fluoride with a larger specific surface area form from the soluble phosphorus and fluoride in PG, slowing down the hydration reaction. The following equations show the relevant reactions. The heat release in the hydration deceleration period also increases with PG content. This is because PG provides an ample supply of SO_4_^2−^, activating the active silico–alumino substances in the matrix and generating hydration products such as C-S-H and ettringite.

(28) $$2F^{-}+Ca^{2+}\to \mathrm{Ca}F_{2}↓$$

(29) $$P_{2}O_{5}+6OH^{-}+3Ca^{2+}\to Ca_{3}(PO_{4}{)}_{2}↓+3H_{2}O$$

To activate the cementitious activity of FA and steel slag, Shen et al. [94] modified FA–SS-based cementitious materials using PG and prepared road base materials. The research results indicated that the PG–FA–SS system had a compressive strength that was 6.9 MPa higher than the FA–SS system at 28 days and satisfied the Chinese standards for semirigid road base material. Moreover, its splitting strength, rebound modulus, and water stability matched or exceeded those of typical road base materials. PG introduced additional calcium ions and sulfate ions into the reaction system, activating the inert silico–aluminous minerals in the system. The interaction of PG, FA, and SS produced large amounts of gel products and ettringite. The pores in the system were filled by rod-shaped ettringite crystals, making the matrix denser.

Usually, PG is an acidic industrial solid waste [98]. However, after special treatment (such as washing or adding lime), PG exhibits alkaline properties with a pH value ranging from 8 to 12. To investigate the impacts of acidic and alkaline PG on the hydration performance of cementitious materials, Costa et al. [95] studied the effects of PG with different pH values on the OPC hydration process. The study showed that acidic PG and the soluble phosphates in PG extended the induction period of hydration and reduced the cumulative heat release, delaying the hydration reaction. It also indicated that fluorides in PG were not the cause of the delayed OPC hydration. The hydration products and mechanical properties of the alkaline PG–OPC system were similar to those of the natural gypsum–OPC system, indicating that alkaline PG had a minor influence on cement hydration. Furthermore, the 28-day compressive strength of the alkaline PG–OPC system was significantly higher than that of the natural gypsum–OPC system.

Phosphate solution often exists in the interstices or microcracks of PG crystals. Additionally, due to isomorphic substitution, P^5+^ replaces S^6+^ in SO_4_^2−^, causing phosphate impurities to dissolve in PG [109]. To address these issues, Yuan et al. [96] studied the impact of PG on OPC hydration performance at different calcination temperatures. The study showed that high-temperature calcination could fully utilize the sulfate activation effect of PG in cementitious materials, enhancing the early strength of the matrix (the early hydration time was advanced by 17–21 h). Furthermore, high-temperature calcination effectively removed impurities such as phosphates and fluorides from PG. Impurity components in PG reduced the contact area between reactants, delaying the induction period and slowing down the hydration reaction. Meanwhile, high-temperature calcination induced a phase transformation in PG. P^5+^ diffused from the surface to the interior of PG, forming PO_4_ by replacing S^6+^, increasing the structural defects in CaSO_4_ crystals, and enhancing PG’s hydration reactivity. The improved reactivity of raw materials in the matrix facilitated the development of the mechanical properties.

**Figure 9:**
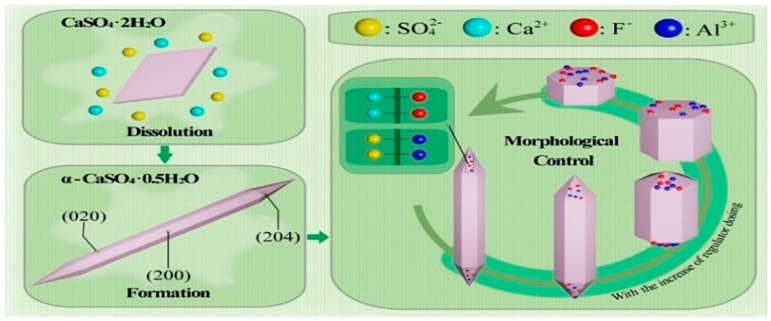
Mechanism of the conversion of PG into α−HH crystals in H_3_PO_4_−H_2_O solution [99].

The autoclave method and the pressurized hydrothermal method are the main commercial production methods for α-HH, and they make its preparation cost higher [110]. Therefore, atmospheric solutions (salt, alcohol, and acid) have become increasingly popular [93]. Chen et al. [99] successfully prepared α-HH from PG in an H_3_PO_4_–H_2_O solution at room temperature, which made PG a value addition in cementitious materials. The research results indicated that all indicators of α-HH met the requirements of the Chinese standard JC/T 2038-2010 [111]. The compressive strength after 2 h of hydration increased by 45%. Meanwhile, the standard consistency and initial/final setting times of the product were optimized (as shown in Table 3). The additives and the H_3_PO_4_–H_2_O solution slowed down the collision nucleation rates of Ca^2+^ and SO_4_^2−^, postponing the nucleation of α-HH in the crystal induction period. In addition, F^−^ and Al^3+^ adsorbed selectively on the end faces of α-HH crystals, hindering the growth of α-HH along the c-axis crystal plane in the generation phase. Changes in the crystal structure during the induction and growth periods ultimately resulted in the transformation of α-HH into short columnar shapes (as shown in Figure 9).

### 3.2. Stabilization of Hazardous Elements

In recent years, the environmentally friendly disposal of BPG has become a hot and challenging issue in various countries. The complex composition of BPG, combined with the presence of heavy metal elements, leads to its substantial accumulation [91,112]. It is noteworthy that cementitious materials have great potential for the environmentally friendly disposal of industrial solid waste [113]. An increasing number of scholars are using the synergistic effects of BPG with other industrial solid wastes to prepare cementitious materials, providing an effective solution and direction for the green disposal of BPG. Once this method is industrialized, it not only effectively solidifies hazardous elements in BPG but also enables the large-scale and value-added utilization of BPG and other industrial solid wastes.

Li et al. [81] used DG to modify RM-based cementitious materials. The study found that DG had a positive impact on the mechanical and environmental performance of the matrix. The leachate of the cementitious material met the Chinese drinking water standards (GB5749-2006) [114]. Subsequently, DG-based cementitious materials were used to prepare road base materials. Practical applications showed that the leachate of the road base material met ion leaching and sewage discharge standards. Zhang et al. [83] used DG to modify CCS-based cementitious materials. The study showed that the leachate of this cementitious material met the standards for drinking water. Additionally, modified cementitious materials were used to prepare stabilized layer materials and underwent a radioactivity test. The test results showed that the exposure index and external exposure index of the material were both less than 1, which complies with the Chinese construction material usage standards (GB 6566-2010) [115]. Chen et al. [47] studied the environmental performance of cementitious materials based on TG. They discovered that TG increased the ettringite level in the matrix, which effectively solidified heavy metal ions in the system. Leachate tests showed that the content of heavy metal ions (such as Cu, As, Co, Mn, Cr, and Sb) met the requirements of GB 16889-2008 [116]. Li et al. [117] used TG to modify coal gangue–aluminum powder–CCS–CSA-based cementitious materials and studied their environmental performance. The research results showed that the abundant ettringite in the system solidified heavy metal elements (Cr, Mn, Zn, Cu, Ni, As, Cd, and Pb). PG contains trace amounts of heavy metals and radioactive elements, hindering its large-scale application and causing harm to the environment. To address these issues, Meskini et al. [118] utilized the solidification effect of cementitious materials on hazardous elements. They prepared PG, FA, and quicklime as cementitious materials for road base materials. The study results showed a significant optimization of the radioactivity index and heavy metal solidification rate of PG. This was due to the roles of chemical solidification and physical encapsulation played by ettringite and gel products in the matrix.

**Figure 10:**
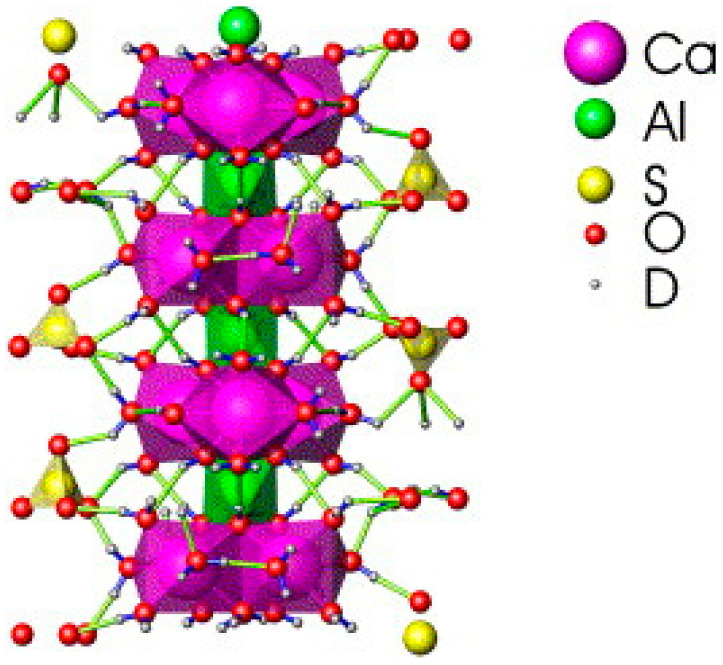
Structure of ettringite [125].

**Figure 11:**
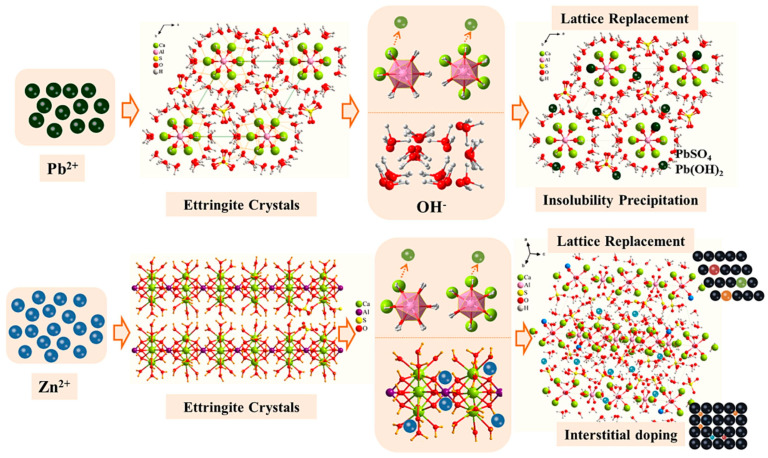
The solidification mechanism of ettringite for heavy metals [69].

Ettringite (Ca_6_Al_2_(SO_4_)_3_(OH)_12_·26H_2_O) is a primary hydration product in gypsum-based cementitious materials. It not only enhances the mechanical properties of the matrix but also has the ability to immobilize oxyanions and heavy metal cations [70]. DG provides an abundant supply of Ca^2+^ and SO_4_^2−^ in cementitious systems, accelerating the rate of ettringite formation and increasing its content. Reports say that the ettringite structure consists of [Ca_6_[Al(OH)_6_]_2_·24H_2_O]^6+^ columns, which are parallel to the c-axis, with [Al(OH)_6_]^3−^ octahedra connected to three Ca^2+^ neighbors and H_2_O molecules completing the coordination polyhedra [119,120]. Ca^2+^ is coordinated by four OH^−^ groups and four H_2_O molecules. SO_4_^2−^, in the form of [(SO_4_)_3_·2H_2_O]^6−^ inside the channels, connects through hydrogen bonding, as shown by the bright blue dotted lines in the ettringite structure of a typical elongated hexagonal prismatic crystal in Figure 10. This unique structure lets the sulfate ions in ettringite be replaced by oxyanions with similar structures and radii, such as chromate, arsenate, vanadate, and selenate, under specific conditions [121,122,123]. Studies have shown that early hydration of the matrix in the TG–CSA system plays a crucial role in the stable immobilization of heavy metals, especially in controlling the leaching of Cr from the system [117]. The spatial structure composed of gel phases such as ettringite acts as a low-permeability barrier, preventing direct contact between heavy metals and the aqueous solution and, thereby, controlling the migration and leaching of heavy metals [124]. Additionally, some heavy metals can react with other reactants in the matrix to form precipitates [124]. Figure 11 illustrates the mechanism of the immobilization of heavy metals by ettringite during the hydration process.

Simultaneously, ettringite can also stabilize and immobilize divalent or trivalent heavy metal cations. These metal cations can substitute for Al^3+^ in the octahedral sites. Bentorite (Ca_6_(Cr(OH)_6_)_2_(SO_4_)_3_·26H_2_O) is a naturally occurring ettringite where Cr^3+^ substitutes for Al^3+^. Albino et al. [126] conducted experiments on the immobilization of heavy metal cations by ettringite and successfully replaced Al^3+^ with Cu, Cr, Cd, Fe, Pb, and Zn. However, not all situations lead to the stable immobilization of heavy metal ions by ettringite. Successful immobilization conditions include the pH of the cementitious material, the type of industrial solid waste in the cementitious material, curing temperature, competitive effects between ions, and the extent of carbonation during the curing process, among others [127].

**Figure 12:**
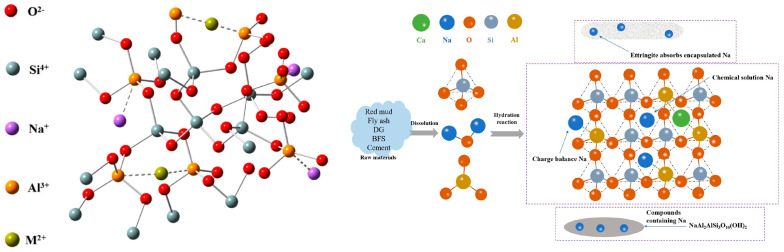
The mechanism of hazardous element solidification in DG−based cementitious materials [81,128].

The hydration process in cementitious materials essentially involves the transition of [Si(Al)O_4_] tetrahedra from a polymerized state to an isolated state and back to a polymerized state. Under specific conditions, Al replaces Si in the [SiO_4_] tetrahedra, creating [AlO_4_] tetrahedra. These tetrahedra form a three-dimensional network structure by connecting through bridging oxygen bonds. Because the negative charge from Al replaces Si in [AlO_4_] tetrahedra, monovalent or divalent cations in the matrix, such as Na, As, Cr, and Pb, are drawn to the vacancies in the tetrahedral sites via charge balance effects. This achieves the bonding and immobilization of hazardous elements, as shown in Figure 12.

Additionally, BPG-based cementitious materials can immobilize hazardous ions from raw materials through physical encapsulation and adsorption. DG accelerates the growth rate of hydration products in the cementitious material, promoting the formation of dense network structures of hydration products such as ettringite, C-S-H, and C-A-S-H. This unique structure envelops heavy metal or harmful ion clusters within the cementitious phase, accomplishing the physical encapsulation of hazardous elements. The adsorption of hazardous ions mainly occurs on the surface of the gel phase, which has numerous micropores and strong adsorption capabilities for hazardous ions such as sodium, arsenic, chromium, and lead due to its large specific surface area. The hazardous elements in BPG-based cementitious materials are effectively stabilized and solidified through methods such as chemical bonding, physical encapsulation, and surface adsorption.

**Figure 13:**
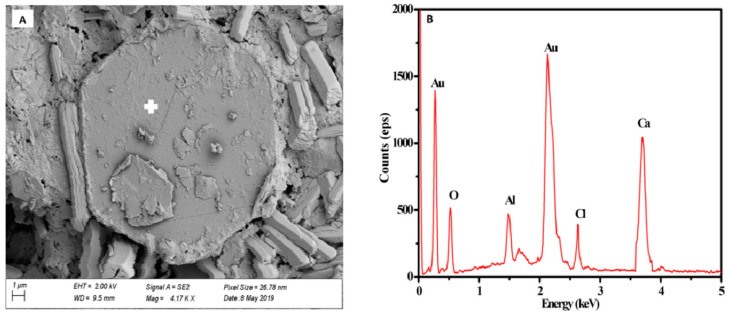
Hexagonal plate-shaped Friedel’s salt, (**A**): SEM; (**B**): EDS [86].

It is worth noting that the formation of new phases in BPG-based cementitious materials is accompanied by the immobilization of harmful elements. Li et al. [86] utilized BFS and CAC as additives in modified DG-based cementitious materials. The study revealed an enhanced ability to immobilize Cl^−^ in the DG–BFS–CAC system. This improvement is attributed to the synergistic effects in the system, which promote the formation of chloride-containing phases (such as Friedel’s or Kuzel’s salt). Figure 13 shows the hexagonal plate-shaped Friedel’s salt formed in this system after 28 days of curing.

## 4. Discussion

(1) The impact of BPG on the mechanical properties of cementitious materials

**Figure 14:**
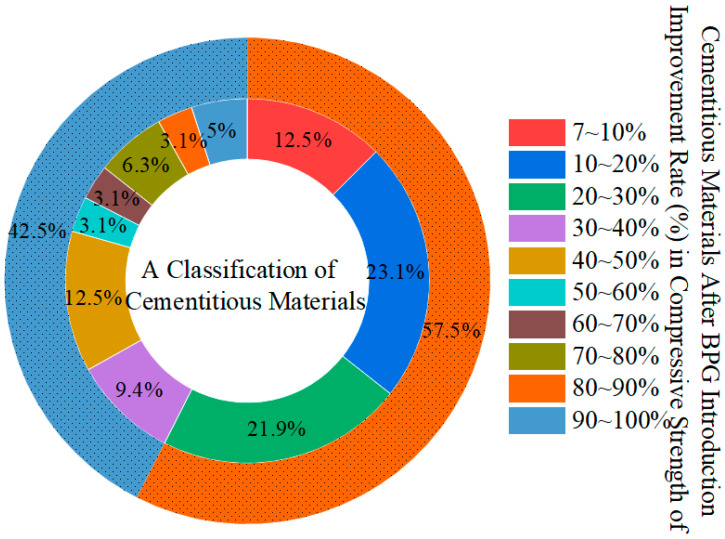
Classification chart based on compressive strength growth rate of cementitious materials after adding BPG.

An appropriate amount of BPG can enhance the mechanical properties of cementitious materials. BPG promotes the formation of ettringite during the hydration process, and its content is directly proportional to the amount of BPG added. The expansive properties of ettringite compensate for the reduction in volume of cement-based materials during hydration. Furthermore, BPG promotes the formation of a high-strength hydration product, which is the 5·1·7 phase, in magnesium–oxysulfate-cement-based cementitious materials. Most importantly, an appropriate amount of AFt fills the micro-pores that develop in cementitious materials, resulting in a denser matrix structure. Big data analysis shows that adding BPG can increase the compressive strength of cementitious materials by approximately 7–30% under suitable conditions (as shown in the Figure 14).

The formation of AFm has a negative impact on the mechanical properties of BPG-based cementitious materials. Appropriate BPG inhibited the formation of AFm (calcium sulfoaluminate monosulfide) in the later stage of the hydration reaction. AFm formation occurs due to the transformation of tetrahedral coordinated aluminum into octahedral coordinated aluminum, which reacts with Ca^2+^ and SO_4_^2−^. AFm can easily be converted into AFt under certain conditions. Due to the different densities of AFt and AFm, excessive AFm formation can cause changes in the matrix volume, disrupting the matrix structure. Additionally, BPG-based cementitious materials should not be exposed to high-temperature environments. This is because high-temperature environments (above 65 °C) promote the formation of AFm during the early stages of hydration and induce the conversion of AFt into AFm during the later stages of hydration.

Hazardous elements in BPG delay the progress of hydration reactions, thereby impairing the mechanical properties of the material. These elements attach to the surface of the hydration products, hindering their effective contact area and leading to an extended induction period and reduced cumulative heat release. Excessive BPG reduces the water resistance of cementitious materials. This is because unreacted gypsum particles dissolve rapidly in water, which disrupts the matrix structure and lowers the mechanical properties.

The main impurity in DG is CaCl_2_, which causes severe corrosion in construction-related cementitious materials. TG contains Fe^3+^ impurities, which introduce amorphous Fe(OH)_3_ into the hydration reaction system. Fe(OH)_3_ encapsulates hydration products and excessive amounts reduce the hydration performance. PG’s primary impurities consist of soluble phosphorus and fluoride. These impurities encapsulate raw materials during the reaction, hindering the progress of hydration reactions.

(2) Synergistic effects of BPG and other industrial solid wastes in cementitious materials

BPG enhances the pozzolanic activity of silico–aluminous solid wastes. Silico–aluminous solid wastes such as BFS, SS, and FA contain a significant amount of silico–aluminous minerals, but these components are not inherently active. Due to its composition, BPG exhibits a superior sulfate activation effect on silico–aluminous solid wastes. The Ca^2+^ and SO_4_^2−^ in BPG react with SiO_4_^4−^ and AlO_4_^4−^ in silico–aluminous solid waste, forming hydrated calcium silicate (C-S-H) and ettringite. Additionally, alkali metal salts (potassium and sodium salts) in BPG accelerate the hydration reactions.

Silico–aluminous solid waste optimizes the hydration and environmental performance of BPG-based cementitious materials. In the early stages of hydration, silico–aluminous solid waste accelerates sulfate consumption, promoting the formation of AFt and suppressing the formation of AFm in the later stages of hydration. The reduced AFm content stabilizes the matrix structure. Silico–aluminous solid waste also reacts with impurities in BPG (Cl^−^, Fe^3+^, soluble phosphorus, and fluoride), forming insoluble substances (Ca_4_Al_2_(OH)_12_Cl_2_·4H_2_O, Ca_3_(PO_4_)_2_, CaF_2_, CaSiF_6_, and Ca_6_Fe_2_(SO_4_)_3_(OH)_12_·26H_2_O, Ca_6_[Al_l1_-xFe_x_(OH)_6_]_2_(SO_4_)_3_·26H_2_O) and facilitating the hydration reactions. The generated insoluble minerals fill the voids between the matrix structures, thereby increasing the density of the matrix.

There are synergistic effects among BPG, silico–aluminous solid wastes, and alkaline wastes. Industrial solid wastes such as RM and CCS contain a significant amount of alkali, making them unsuitable for the preparation of cementitious materials alone. BPG, combined with alkaline waste materials (exhibiting alkali activation effect), accelerates the leaching of silico–aluminous minerals (SiO_2_ and AlO^2−^) in silico–aluminous solid wastes, accelerating the hydration process. Additionally, BPG is typically acidic, and it not only neutralizes alkali but also utilizes an exothermic reaction to accelerate the matrix hydration process. Furthermore, BPG compensates for the reduction in volume caused by alkali activation systems.

Silico–aluminous solid wastes and alkaline wastes optimize the hydration and environmental performance of BPG-based cementitious material. These components form ettringite, C-S-H, and C-A-S-H through valuable synergistic hydration reactions, creating a complex and stable three-dimensional network structure. This structure effectively encapsulates unreacted gypsum particles, reducing their dissolution and improving the water resistance of the matrix. Simultaneously, the efficient hydration reaction system accelerates the transformation of impurities into insoluble minerals.

The Graphical Abstract illustrates the synergistic effects among BPG, alkaline industrial solid waste, aluminosilicate industrial solid waste, and cement.

(3) BPG’s Contribution to the Environmental Performance of Cementitious Materials

In BPG-based cementitious materials, the common hydration product is ettringite, which not only enhances the mechanical properties of the matrix but can also immobilize oxyanions and heavy metal cations. SO_4_^2−^ in the ettringite structure is linked by hydrogen bonds, allowing sulfate ions to be replaced by oxyanions with similar structures and radii under specific conditions, such as chromate, arsenate, vanadate, and selenate. Additionally, ettringite can stabilize and immobilize divalent or trivalent heavy metal cations. These metal cations can substitute for Al^3+^ in the octahedral sites of ettringite by, for example, forming compounds such as Ca_6_(Cr(OH)_6_)_2_(SO_4_)_3_·26H_2_O. Furthermore, cementitious materials can chemically bond heavy metal ions. Under specific hydration conditions, Si in the [SiO_4_] tetrahedra can be replaced by Al, generating [AlO_4_] tetrahedra. Due to charge balance effects, heavy metal cations (such as Na, As, Cr, and Pb) can be captured, leading to the immobilization of hazardous elements. Additionally, BPG-based cementitious materials can immobilize hazardous ions through physical encapsulation and adsorption.

It is worth noting that the performance of BPG with other industrial-solid-waste-based cementitious materials is significantly better than that of individual waste-based materials, promoting the proportional use of industrial solid waste in cementitious materials. The increased ratio of industrial solid waste significantly reduces carbon emissions during the preparation of cementitious materials. It has been reported that replacing one ton of raw materials with industrial solid waste in cement preparation can reduce CO_2_ emissions by approximately 700–850 kg [17,18,19,20]. In summary, under specific conditions, BPG-based cementitious materials exhibit excellent environmental performance.

**Figure 15:**
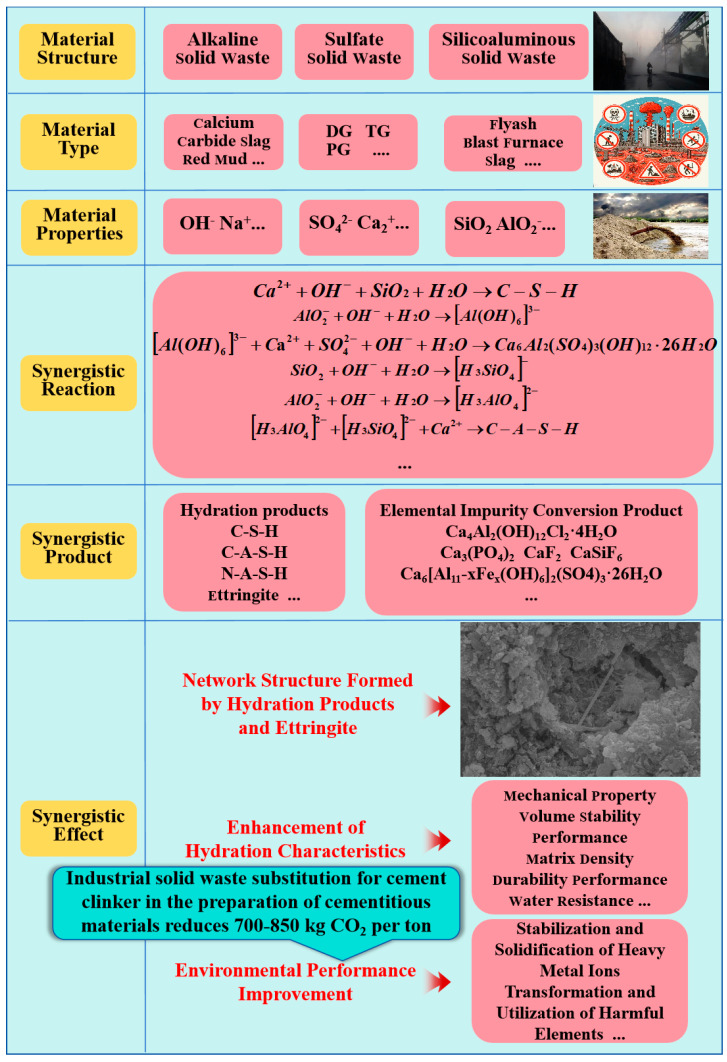
Synergistic effects among BPG, alkaline solid waste, and sulfate solid waste. Note: This figure is a summary diagram and does not involve any references or copyright issues.

The mechanism of the synergistic reactions among BPG, alkaline solid waste, and aluminosilicate solid waste is shown in Figure 15.

Note: Data collected from Web of Science, keywords: flue gas desulfurization gypsum, titanium gypsum, or phosphogypsum, time period: 2021–2023 [129]. In Figure 14, the evaluation principle for the increase in compressive strength is defined as follows: Compressive Strength Increase Ratio = ((BPG + other materials-based cementitious materials) − (other materials-based cementitious materials))/(other materials-based cementitious materials) × 100. Data points with an increase in ratio exceeding 100% were excluded to improve the generality of the data. Subsequently, these data were categorized according to their increase ratios. This figure is a summary diagram and does not involve copyright issues.

## 5. Conclusions and Prospects

### 5.1. Conclusions

(1) A moderate amount of BPG can compensate for the reduction in volume of cement-based cementitious materials caused by alkali activation. Big data analysis shows that under certain conditions, BPG can increase the compressive strength of cementitious materials by about 7–30%. At the same time, BPG can also inhibit the formation of AFm (monosulfate) in the later stage of hydration. In addition, the main impurities in DG, TG, and PG are Cl^−^, Fe^3+^, and soluble phosphorus and fluorides, respectively. These hazardous impurities hinder the hydration reaction.
(2) BPG can enhance the pozzolanic activity of silico–aluminate and alkaline solid wastes. Meanwhile, silico–aluminate and alkaline solid wastes can also increase the reaction rate and degree of BPG during hydration. It is noteworthy that there is a positive synergism among between sulfate solid wastes (BPG), silico–aluminate solid wastes, and alkaline solid wastes. Under specific synergistic effects, the impurity elements in BPG (Cl^−^, Fe^3+^, soluble phosphorus, and fluorides) form insoluble substances to increase the density of the matrix.
(3) The main hydration product of BPG-based cementitious materials is ettringite. Under certain conditions, ettringite, which has a unique structure, can positively solidify heavy metal oxyanions and cations. In addition, BPG-based cementitious materials can also solidify hazardous elements through chemical bonding, physical wrapping, and surface adsorption. Moreover, using industrial solid waste as a raw material for cement production under normal temperatures can significantly reduce the carbon emissions of the cement industry.

### 5.2. Prospects

(1) Through a review of BPG-based cementitious materials, it has been found that BPG combined with alkaline waste and silico–aluminous solid wastes exhibits excellent hydration and environmental performance. This combination could be one of the main future research directions for BPG-based composite cement.
(2) Upon review, it is essential to strengthen computer simulation studies of the hydration process and immobilization of hazardous elements in BPG-based composite cement. This approach is beneficial for unraveling the hazards of the reaction processes in BPG-based cementitious materials, facilitating the green recycling of BPG.
(3) It is imperative to promptly establish a life-cycle assessment system for BPG-based cementitious materials. Analyzing and addressing bottlenecks in BPG utilization from resource and environmental perspectives will expedite the exploration of industrial application models and pathways for BPG.
(4) Based on the distinctive industrial development characteristics of different regions, collaborative utilization pathways between BPG and other industrial solid wastes can be established. This will promote the low-carbon sustainable development of industrial clusters.

## Data Availability

The original contributions presented in the study are included in the article, further inquiries can be directed to the corresponding author.
